# Extrahepatic Biliary Mucinous Cystic Neoplasm as an Uncommon Cause of Acute Cholangitis

**DOI:** 10.7759/cureus.80571

**Published:** 2025-03-14

**Authors:** Gloria A Peña Montañez, Carlos F Gallegos De Luna, Mónica E Carpintero Martínez, Erwin Soc Nicolás, José R Hernández Ortíz

**Affiliations:** 1 General Surgery, Centro Médico Nacional de Occidente, Instituto Mexicano del Seguro Social (IMSS), Guadalajara, MEX; 2 General Surgery, Hospital General de Zona 3, Instituto Mexicano del Seguro Social (IMSS), Aguascalientes, MEX

**Keywords:** biliary tract dilation, cholangitis, extrahepatic biliary cystadenoma, hepaticojejunostomy, mucinous cystic neoplasm biliary

## Abstract

Biliary mucinous cystic neoplasms (BMCNs) are rare tumors that account for less than 5% of cystic lesions in the biliary tract, predominantly affecting middle-aged women and often presenting as asymptomatic or with nonspecific symptoms. The primary treatment is surgical resection with negative margins. We report a case of grade I non-invasive BMCN that was completely extrahepatic, treated with biliary resection and hepaticojejunostomy. A 42-year-old female with a history of type 2 diabetes was referred for multiple episodes of acute cholangitis. Magnetic resonance cholangiography revealed complete occupation of the common bile duct by a multilocular lesion with thin septa extending into the left main bile duct. Biliary resection with Roux-en-Y hepaticojejunostomy was performed. Histopathological examination identified a mucinous cystic neoplasm with no evidence of cellular atypia, invasion, or malignancy. BMCNs are characterized by multilocular cysts lined by mucin-producing epithelium and a subepithelial stroma resembling ovarian tissue, suggesting hormonal dependence and an embryological origin. Preoperative diagnosis relies on imaging studies, with computed tomography being crucial for identifying the distinctive “cyst-in-cyst” appearance with septa. Complete resection is the preferred treatment due to the risk of malignancy, though recurrence remains a challenge in invasive cases. Mucinous cystic neoplasms of the liver are rare lesions, especially those that are extrahepatic, presenting diagnostic and therapeutic challenges. Continued research into this pathology is essential to optimize its management, treatment, and prognosis, particularly in cases of recurrence.

## Introduction

Biliary mucinous cystic neoplasms (BMCNs), previously known as "biliary cystadenoma" and "biliary cystadenocarcinoma," are rare neoplasms that account for less than 5% of all cystic lesions originating in the intra- and extrahepatic biliary tract, with the extrahepatic subtype being the least common (10-15%) [1,2]. These neoplasms most commonly affect middle-aged women and are typically asymptomatic or present with nonspecific signs and symptoms, which makes clinical diagnosis particularly challenging [1,3]. Histologically, BMCNs are multilocular cystic tumors with an ovarian-like stroma, lined by cuboidal or columnar secretory epithelium [4,5]. They are further classified into invasive and non-invasive types. Although BMCNs are generally considered benign, there is a risk of malignant transformation in up to 20% of cases [6]. Non-invasive BMCNs are classified based on the degree of atypia: (I) low-grade intraepithelial dysplasia; (II) intermediate-grade dysplasia; and (III) high-grade dysplasia [1,7]. In general, the treatment of choice is surgical resection with negative margins, with the extent of resection determined by the location of the lesion [1,4].

We present the case of a middle-aged female patient with grade I non-invasive BMCN, entirely extrahepatic, who required biliary resection with hepaticojejunostomy.

## Case presentation

A 42-year-old female patient from Baja California Sur, Mexico, was referred to our unit due to multiple episodes of acute cholangitis. Her relevant medical history included a 14-year diagnosis of type 2 diabetes, managed with metformin and glibenclamide; nulliparity; no use of hormonal contraceptives; no family history of hepatobiliary disease; and no prior surgical history or other clinically relevant factors.

The patient began experiencing symptoms in 2022, with multiple episodes of mild acute cholangitis, requiring hospitalization three times with satisfactory medical management. Suspecting Caroli's disease based on imaging studies, she was referred to our specialized liver and biliary unit for comprehensive evaluation. During the initial assessment, the patient reported intense pruritus, occasional low-grade fever, but no nausea, abdominal pain, or weight loss. Physical examination revealed scleral jaundice and hepatomegaly, without abdominal pain. Laboratory tests showed no changes in the complete blood count, along with hyperbilirubinemia (direct bilirubin 3.2 mg/dL (<0.3 mg/dL), indirect bilirubin 3.5 mg/dL (0.2-1.2 mg/dL)), elevated alkaline phosphatase (ALP) of 339 U/L (44-147 U/L), and γ-glutamyl transferase (GGT) of 535 U/L (6-42 U/L). Tumor markers revealed a slight elevation in CA 19-9 to 151 kU/L (<37 kU/L).

**Figure 1:**
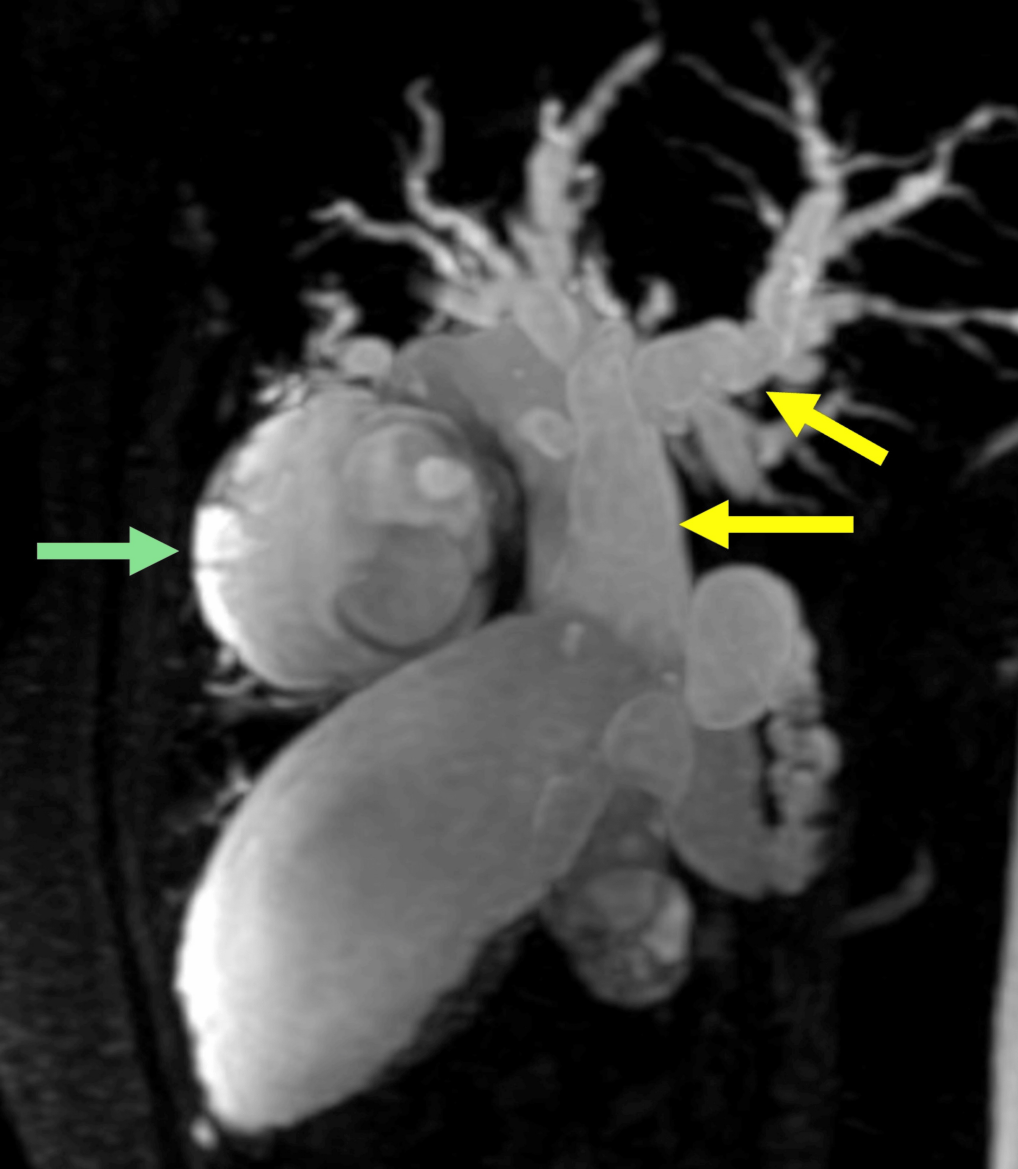
Magnetic cholangiopancreatography Dilation of the intrahepatic and extrahepatic bile ducts is noted. Green arrow: A round mass measuring 4 x 5 x 5.3 cm is noted in the superior portion of segment 4, arising from the second- and third-order left bile ducts. The mass demonstrates heterogeneous signal intensity with thin septa. Yellow arrows: In the region of the common hepatic and common bile ducts, heterogeneous signal intensity is noted, with multiple septa and confluent nodular images of undetermined origin.

**Figure 2:**
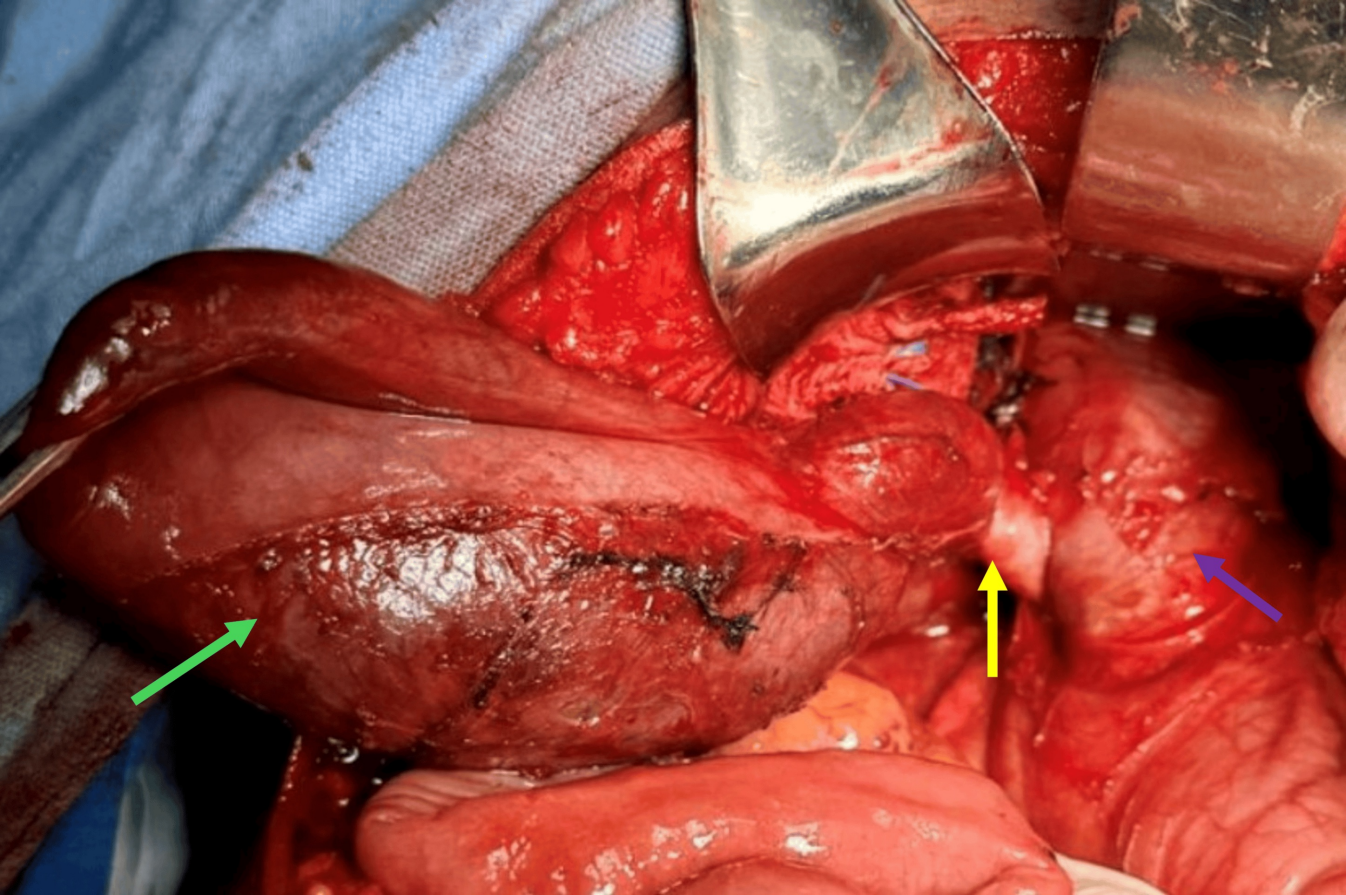
Surgery findings The gallbladder (green arrow), cystic duct (yellow arrow), and common bile duct (purple arrow) dilated up to 40 mm in diameter, with the presence of a mucinous cystic tumor exhibiting an ischemic appearance.

To complement the diagnostic approach, magnetic resonance cholangiography was performed, which revealed complete occupation of the common bile duct by a multilocular image with thin septa, extending into the left main bile duct (Figure 1). The lesion measured 71 x 43 x 44 mm, with choledochal ectasia up to 27 mm, a 20 mm cystic duct, and right and left main bile ducts measuring 20 mm and 44 mm, respectively. Additionally, dilatation of second- and third-order intrahepatic branches of 6 mm was observed, with tortuous pathways. Upon the diagnosis of an extrahepatic biliary cystic lesion, the decision was made to perform biliary resection with Roux-en-Y hepaticojejunostomy (60 cm biliary loop and 60 cm alimentary loop) (Figure 2). The surgical procedure was completed without complications, with findings of a congested and enlarged common bile duct (up to 40 mm in diameter), containing an ischemic-appearing mucinous cystic tumor approximately 60 mm in length and 20 mm in diameter, involving the entire extrahepatic bile duct. The liver appeared atrophic and micronodular, and a liver biopsy was subsequently performed.

**Figure 3:**
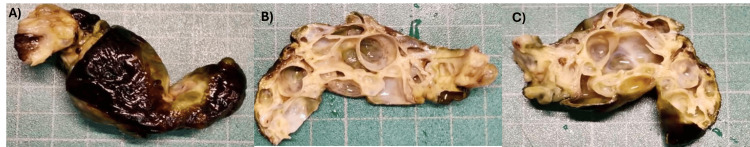
Extrahepatic biliary mucinous cystic neoplasm (A) Surgical specimen of cyst resection. Coronal section of the neoplasm with multiple cysts inside, right side (B) and left side (C). The largest measured up to 3 cm in diameter.

**Figure 4:**
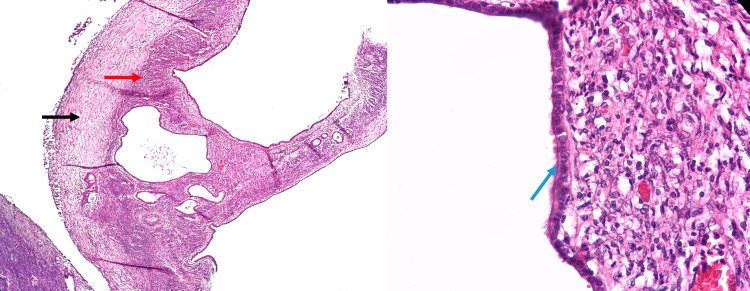
Microscopic view of the sample Microscopy revealed the presence of connective tissue (black arrow), ovarian-type stroma (red arrow), and simple cuboidal biliary-type epithelium (blue arrow), with no evidence of dysplasia or mitosis.

The histopathological examination revealed a biliary cystadenoma or mucinous cystic neoplasm measuring 8.5 x 2.5 cm, with no evidence of cellular atypia, invasion, or malignancy (Figures 3-4). Immunohistochemical staining showed positivity for CK7 and CK19 in the bile duct epithelium. The liver biopsy demonstrated intracellular and intracanalicular cholestasis, ductal proliferation, and the formation of numerous portal-to-portal fibrous septa, consistent with chronic obstruction of the extrahepatic biliary tract. The postoperative course was uncomplicated, and the patient was discharged for home care on day 7. Follow-up at three months revealed laboratory findings of total bilirubin 1.48 mg/dL (0.1-1.2 mg/dL), direct bilirubin 1.25 mg/dL, GGT 340 U/L, ALP 308 U/L, alanine transaminase (ALT) 120 U/L (4-36 U/L), and aspartate aminotransferase (AST) 93 U/L (8-36 U/L). To date, the patient remains free of complications. The projected postoperative follow-up will consist of performing quarterly laboratory tests, including liver function tests and bilirubin levels, to assess the trend toward reduction and ensure that the values remain within normal ranges. Annually, it is projected to request imaging studies, primarily magnetic resonance imaging, to evaluate the permeability of the anastomosis.

## Discussion

Although the first case of biliary cystadenoma was published in 1887 and the first resection was performed in 1892, it was not properly defined until 2010 by the World Health Organization (WHO) as an epithelial cyst-forming neoplasm, frequently without communication with the biliary ducts [7]. It is composed of cuboidal or columnar mucin-producing epithelium and is associated with an ovarian-like subepithelial stroma [7]. The presence of this stroma as a prerequisite for its development, along with the apparent hormonal dependence of these tumors, aligns with their predilection for women in the fifth decade of life. This has led to the theory of an embryological origin, where gonadal cells migrate to the liver surface, stimulating the proliferation of adjacent biliary or pancreatic ducts [1,2,8].

To establish the diagnosis, it is essential to consider that preoperative biopsy is contraindicated due to the risk of peritoneal seeding and subsequent peritoneal carcinomatosis [1]. Therefore, imaging studies should raise suspicion, although their diagnostic accuracy, particularly in distinguishing non-invasive BMCNs from invasive BMCNs, has been reported to be less than 50%. Nevertheless, these studies are valuable for lesion localization and surgical planning [1,9,10]. The main distinguishing feature of the BMCN observed in our patient, which provided evidence for the preoperative presumptive diagnosis and subsequent decision-making, was the appearance of multilocular cysts with thin septa, the characteristic "cyst-in-cyst" image. This finding has reported sensitivity and specificity values of 81% and 95%, respectively, in computed tomography [9,10].

Although no specific markers or laboratory tests consistently identify a BMCN, it is important to consider standard liver cancer markers to exclude tumors with similar presentations. Liver function tests are usually normal, but in cases such as the present one, with intra- or extrahepatic biliary obstruction, elevated levels of bilirubin, ALP, GGT, and CA 19-9 have been recorded [5,9].

Histologically, a constant and defining characteristic of BMCNs is the presence of an ovarian-like subepithelial stroma, with its characteristic immunological profile (estrogen receptor, progesterone receptor, and α-inhibin), regardless of the epithelial lining, which is predominantly biliary (80%) and, to a lesser extent, intestinal (20%). This lining consists of a single layer of cuboidal or columnar epithelial cells resting on a basal membrane and is similar to the epithelium of the gallbladder or biliary ducts [5,9]. This epithelial lining exhibits a specific phenotype, with immunoreactivity to CK7, CK19, and MUC1, and additionally expresses MUC2 and MUC5AC in borderline and malignant cases [5,9].

Complete resection is the preferred treatment whenever feasible, due to the risk of malignant transformation of the epithelial lining over time. Partial excision is often associated with higher recurrence rates and a less favorable prognosis compared to complete resection, especially in invasive BMCN cases, where recurrence rates of approximately 10% have even been reported after complete resection [1,8]. There are no large case series or extensive long-term follow-up reports, particularly for patients with an extrahepatic BMCN. Therefore, although the short-term prognosis is favorable, the long-term outcome for our patient remains uncertain.

## Conclusions

Mucinous cystic neoplasms of the liver are uncommon lesions that present a diagnostic and therapeutic challenge, particularly in cases where they are entirely extrahepatic, as they can easily be confused with other entities. Due to their malignant potential, the treatment of choice remains complete surgical resection of the lesion, whether by laparoscopic or open approach. It is essential to continue researching this pathology to adequately characterize the approach, treatment, and prognosis, especially in cases of recurrence.
